# Facile band gap tuning in graphene–brucite heterojunctions

**DOI:** 10.1038/s41598-023-50037-z

**Published:** 2023-12-28

**Authors:** Gianfranco Ulian, Giovanni Valdrè

**Affiliations:** Dipartimento di Scienze Biologiche, Geologiche e Ambientali, Centro di Ricerche Interdisciplinari di Biomineralogia, Cristallografia e Biomateriali, Università di Bologna “Alma Mater Studiorum”, Piazza di Porta San Donato 1, 40126 Bologna, Italy

**Keywords:** Mineralogy, Electronic properties and materials, Surfaces, interfaces and thin films, Density functional theory

## Abstract

The zero band gap of pure graphene is a well-known issue that limits some specific applications of graphene in opto- and microelectronics. This led to several research studies in the so‐called van der Waals composites (known as heterostructures, or heterojunctions), where two monolayers of different materials are stacked and held together by dispersive interactions. In this paper, we introduced and considered a single layer of brucite Mg(OH)_2_, an overlooked 2D material that can be easily produced by exfoliation (like graphene from graphite), for the creation of the heterojunction. First principles simulations showed that brucite/graphene composites can modify the electronic properties (position of the Dirac cone with respect to the Fermi level and band gap) according to the crystallographic stacking and the presence of point defects. The present work represents then an important step forward in understanding and finding new ways to design two-dimensional materials with tailored electronic and physical properties.

## Introduction

It is well known that graphene, a single layer of graphite firstly produced by Geim and Novoselov with micromechanical exfoliation^1^, presents a peculiar electronic band structure, because electrons that move through this 2D material behave as massless Dirac fermions, i.e., they show a linear relation between energy and momentum^2,3^. However, the presence of the Dirac point, namely the K-point in the first Brillouin zone where the valence band touches the conduction one, means that pristine graphene has an almost zero concentration of charge carriers^4^. Hence, graphene is a zero band gap semiconductor, which exhibits a metallic behaviour if the Fermi energy is tuned by applying a gate voltage^2,5^. Furthermore, different many-body interactions, for instance electron–electron and electron–phonon, have significant effects on the band structure of graphene^6,7^. These discoveries, together with characteristic transport phenomena (e.g., electron mobility up to about 10^6^ cm^2^ V^–1^ s^–1^ for suspended samples) and optical properties, led to significant efforts in finding advanced applications of graphene and gave rise to many studies of other 2D materials, for instance transition metal dichalcogenides (e.g., MoS_2_, WS_2_, and MoSe_2_)^8,9^. One of such applications is the realization of solid-state electronic devices^10^, in particular field effect transistors (FETs), where two-dimensional materials could surpass the metal oxide semiconductor technology and extend the Moore’s law^11,12^. In general, a FET is made of a conductive surface channel connected to source and drain electrodes, and a gate electrode separated from the channel by an insulating layer (called dielectric). The aim is controlling the current flow along the channel when a voltage is applied to the gate.

However, two main issues remain unresolved for the employment of pristine graphene in these specific applications, i.e., (i) the absence of a band gap and (ii) the type of interaction between graphene and the substrate. Opening the band gap of graphene is highly desirable to make graphene a semiconductor, which is of utmost importance for optoelectronic devices. One of the possible solutions to this problem is creating stacks of different 2D materials, where the layers are held together by weak van der Waals (vdW) interactions. These stacks can be made from two to several layers (multi-layers), resulting in composite structures called heterostructures of heterojunctions^13,14^. The properties of these composite materials can be tuned by different parameters, for example the type of 2D materials employed, the number and the stacking order of the layers (e.g. AB-AB, AAB-AAB), and their spatial orientation relative to each other^13,14^. Hence, using the words of Kroemer, the heterostructures presenting specific interfaces between different materials become the device^15^.

Indeed, it is known that breaking the translation symmetry could result in strong alterations of the electronic, optical, and magnetic properties of the materials, leading also to many interesting phenomena^16^. However, the carrier mobility of graphene, one of the properties of interest in FETs, may be deeply affected (lowered) by the graphene-to-substrate interactions. In this context, while muscovite mica is one of the typical substrates for the analysis of graphene and 2D materials in general^17,18^, *α*-quartz SiO_2_ surfaces, especially the (001), are preferentially used for the gate insulating material in the graphene-based nanoelectronic devices^19^. The (001) surface of *α*-quartz is typically hydroxylated due to reaction with atmospheric water, thus it exposes both highly polarized silanol groups (Si–OH) that present a complex surficial morphology due to the network of O–H–O hydrogen bonds, and Si–O–Si (siloxane) showing a weak polarization (see for instance the very detailed review of Rimola and co-workers on this topic^20^). In addition, it was demonstrated that the carrier mobility in graphene may be reduced because of the presence of scattering centres at the substrate, e.g., corrugations and (charged) defects^19,21^. It follows that the orientation of the silanol groups in the (001)* α*-quartz surface is not optimal for this kind of applications. Hence, there is still a need of reliable 2D materials with both good hydrophobicity and surface flatness, unavoidable requirements to create a suitable carrier mobility in graphene-based heterostructures for the development of nano-electronic devices.

**Figure 1:**
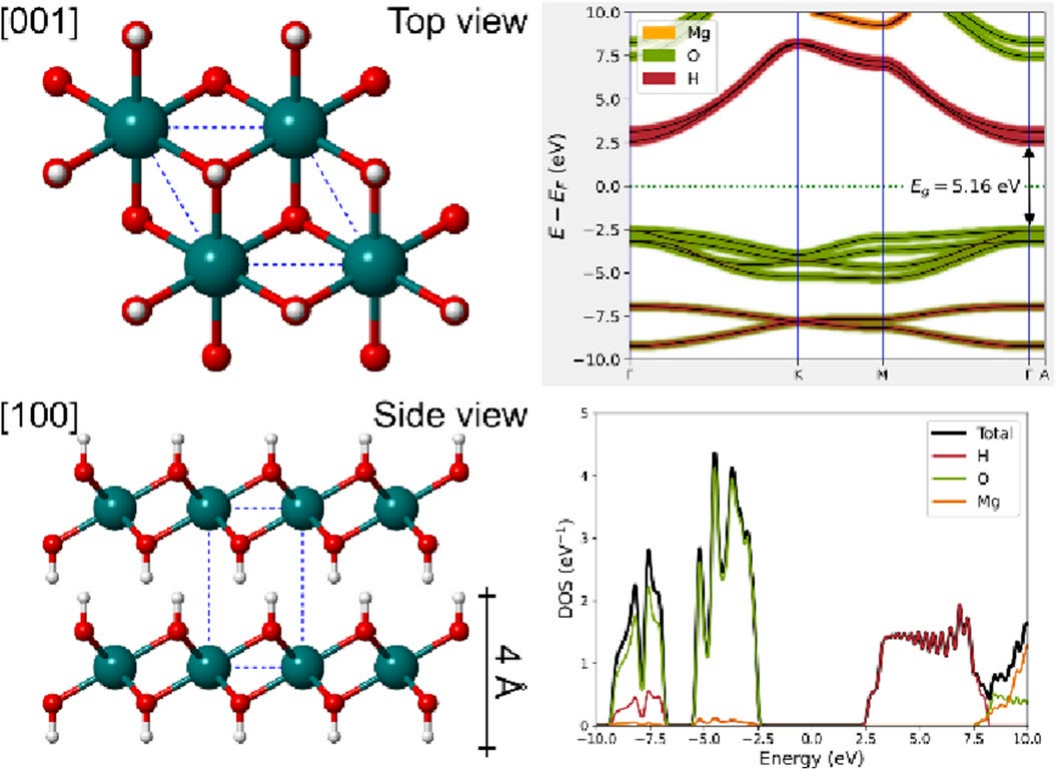
Crystal structure and electronic properties of magnesium hydroxide, Mg(OH)_2_ (brucite). On the left, two views (top and side) of the unit cell of brucite (space group $P\overline{3 }m1$), showing the layered nature and the thickness of a single sheet of about 4 Å. Magnesium, oxygen and hydrogen atoms are coloured in dark cyan, red and white, respectively. On the right, the electronic band structure (upper panel) and the density of states (lower panel, total and atom-projected) of the monolayer of Mg(OH)_2_ obtained from PBE-D2 simulations are reported.

In this context, there exist natural crystals (minerals) presenting both high surface flatness and hydrophobicity, such as magnesium hydroxide Mg(OH)_2_, which is also known as “brucite”. This mineral, whose graphical representation is shown in Fig. 1, is made of layers of Mg^2+^ ions octahedrally coordinated by hydroxyl groups, stacked along the [001] crystallographic direction and held together by weak long-range interactions. It is an insulating material with a wide band gap^22^, and strongly anisotropic elastic^23^ and optical properties^22^. Differently from quartz, the hydroxyl ions in brucite are regularly aligned along the [001] direction, forming an atomic-flat surface. In addition, brucite is widely available, low cost, non-toxic, easily cleavable. Furthermore, the single layer of Mg(OH)_2_ is also found isolated within the structure of an important layered mineral called clinochlore^24,25^. Kelvin probe force microscopy studies revealed that the brucite-like layer of clinochlore present a hydrophobic behaviour, despite the presence of a high number of OH groups^25^.

In the present work, we performed a detailed ab initio Density Functional Theory investigation at the PBE-D2 level and using atom-centred atomic orbitals to characterize the interaction between a single (001) brucite layer and graphene, and its effects on the structure and electronic properties. The crystallographic relationships between the two monolayers were considered by realizing several heterojunctions to properly account for inequivalent stacking and to investigate how the crystal-chemistry and point defects/substitutions could affect the position of the electronic bands, their occupation, and the emergence of possible band gaps.

## Results

### Crystallographic considerations and smallest brucite/graphene interfaces

We briefly introduce the symmetry of graphene and brucite Mg(OH)_2_, as it is fundamental for modelling the interface between the two materials. The former belongs to the $P\overline{6 }m2$ space group, hexagonal crystal system, which is a subgroup of $P{6}_{3}/mmc$, the space group of graphite (holohedral hexagonal space group). Brucite Mg(OH)_2_ has instead a trigonal crystal structure, with both the bulk and the (001) surface described by the $P\overline{3 }m1$ space group. Alongside this common structure, with ordered hydrogen atoms, there exists a proton-disordered brucite where the O–H bonds are slightly canted with respect to the **c**-axis, leading to a lower symmetry (space group $P\overline{3 }$)^22^. The electrostatic potential of the stoichiometric (001) Mg(OH)_2_ surface is given by triangular patterns of positive and negative potential on the H and O atoms, respectively (see Fig. S1a,b in the Supplementary Materials for a graphical representation). Primitive hexagonal and trigonal crystal systems have lattice parameters *a* = *b* ≠ c and *α* = *β* = 90° and *γ* = 120°. From group theory, $P\overline{6 }m2$ and $P\overline{3 }m1$ space groups have a subgroup in common, s.g. $P3m1$, differing from that of brucite by the lack of inversion centre. For both graphene and brucite, lowering the symmetry to $P3m1$ leads to a reduction of the point group, i.e., the subgroup is non-isomorphic (*translationengleiche subgroup*).

**Figure 2:**
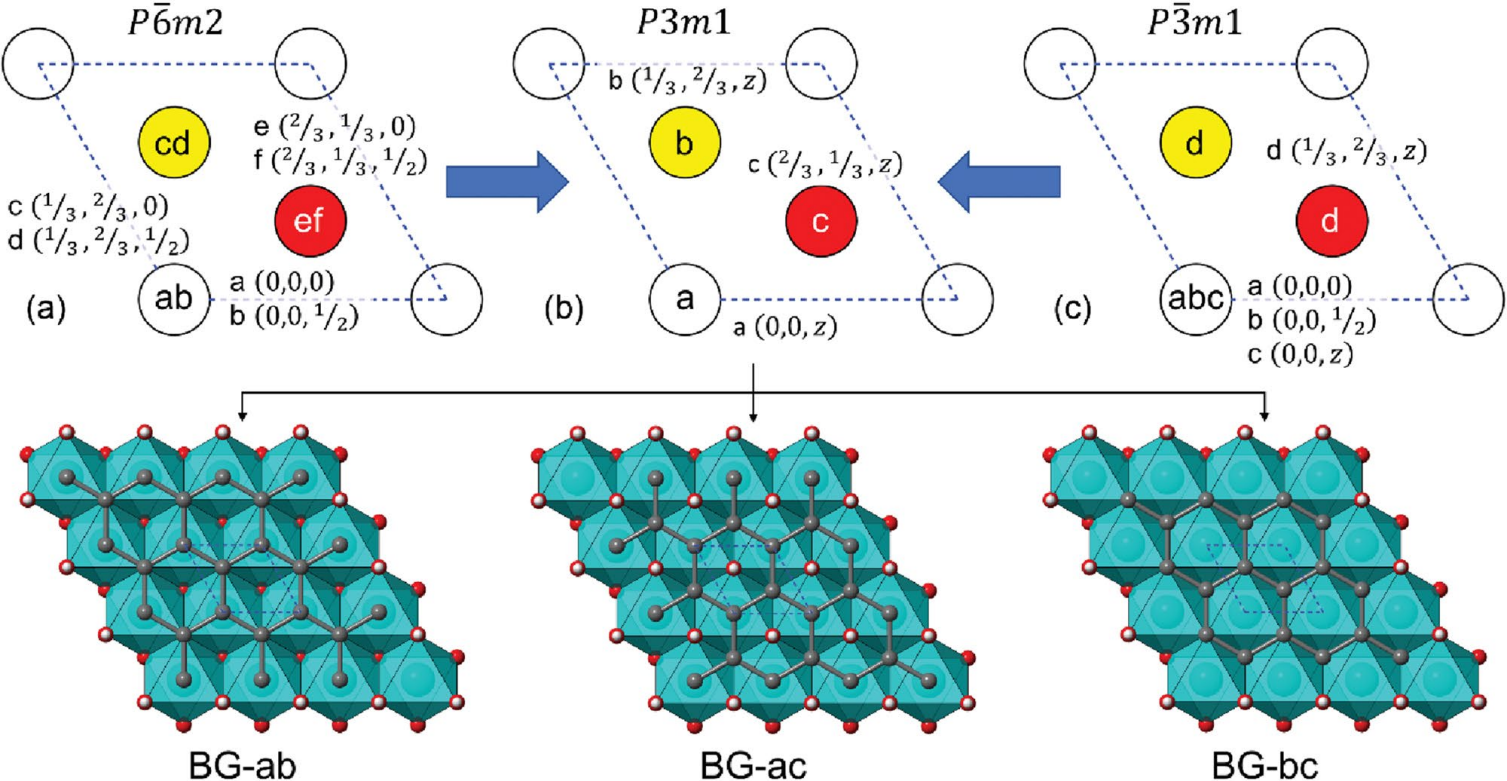
Crystallographic relations between graphene, brucite and bruphene space groups. In the upper panel, the hexagonal/trigonal lattices of graphene (panel **a**, s.g. $P\overline{6 }m2$) and brucite Mg(OH)_2_ (panel **c**, s.g.$P\overline{3 }m1$) are shown, together with their common subgroup $P3m1$ (panel **b**) that is the space group of brucite/graphene (“bruphene”) heterojunction. The graphs are projections along the [001] direction. White, red and yellow circles highlight high-symmetry (Wyckoff) sites, with their labels inside the shape. Note that sites with more than one letter (e.g., ‘a’ and ‘b’ in the circle) indicate Wyckoff positions at different heights (*z* fractional coordinate) in the crystallographic cell. In the lower panel, the three smallest, non-equivalent graphene/brucite (BG) models with $P3m1$ symmetry, as viewed from the [001] direction. The blue dashed lines represent the unit cell of the heterojunction.

Thus, it is in principle possible to perfectly couple (i.e., stack) a single graphene layer to a Mg(OH)_2_ one by placing the atoms in the related crystal sites (Wyckoff positions). This would lead to the smallest brucite/graphene (BG, “bruphene”) crystallographic system, with just seven atoms in the (001) surface structure (heterojunction). As shown in Fig. 2, we could have three different configurations of graphene on brucite with $P3m1$ symmetry, which were labelled as (i) BG-ab, (ii) BG-ac and (iii) BG-bc, where the letters after the hyphen indicate the two Wyckoff positions occupied by the carbon atoms of graphene. The difference between the models can also be seen as which atom of brucite is at the centre of the hexagonal ring of carbon atoms: an oxygen (BG-ab), a hydrogen (BG-ac) or a magnesium atom (BG-bc). However, the slightly lower *a* = *b* lattice parameters of graphene (*a* = 2.466 Å) with respect to that of brucite (*a* = 3.150 Å^26,27^) necessarily leads to strained brucite and graphene layers in the heterostructure, with a mean absolute strain of about 8%. After relaxing the structure of the brucite/graphene (001) heterojunction, we obtained *a* = 2.7118 Å, *a* = 2.7115 Å, and *a* = 2.7116 Å for BG-ab, BG-ac and BG-bc models, respectively (see Table 1). The energy of the three structures is also almost similar, with the BG-ac interface presenting the lowest energy compared to the BG-ab (+ 2.0 meV) and the BG-bc (+ 2.2 meV) ones. The binding energy was calculated as ∆*E* = *E*_BG_ – (*E*_B_ + *E*_G_) ≈ – 12.4 kJ/mol, with *E*_BG_, *E*_B_ and *E*_G_ the energy of the BG heterostructure, the (001) brucite surface and graphene, respectively. This negative value, net of the high energy required to deform the two layers, means that brucite and graphene are favourably held together. However, the distance between the graphene layer and the brucite one, called *d*, is the same (2.486 Å) for the BG-ab and BG-bc models, whereas a slightly shorted separation was observed for the BG-ac system (2.341 Å).

**Table 1:** Lattice parameter *a* = *b*, electronic band gap *E*_*g*_, and work function *W* calculated for graphene, for single-layered (001) brucite and for the different brucite-graphene heterostructures reported in this work.

Model	*a* (Å)	*E*_*g*_ (eV)	*W* (eV)
Graphene	2.466	0	– 4.622
(001) brucite	3.134	5.160	– 3.013
BG-ab	2.712	0	– 4.827
BG-ac	2.712	0	– 4.850
BG-bc	2.712	0	– 4.828
BG-L-c01	5.055	0	– 4.752
BG-L-c02	5.055	0	– 4.752
BG-L-c03	5.055	0	– 4.752
B(Al)G-L-b01	5.035	0	– 4.200
B(Al)G-L-b02	5.035	0	– 4.199
B(Al)G-L-b03	5.035	0	– 4.196
B(Al)G-L-t01	5.024	0.037	– 4.694
B(Al)G-L-t02	5.032	0.042	– 4.706
B(Al)G-L-t03	5.031	0.008	– 4.713

**Figure 3:**
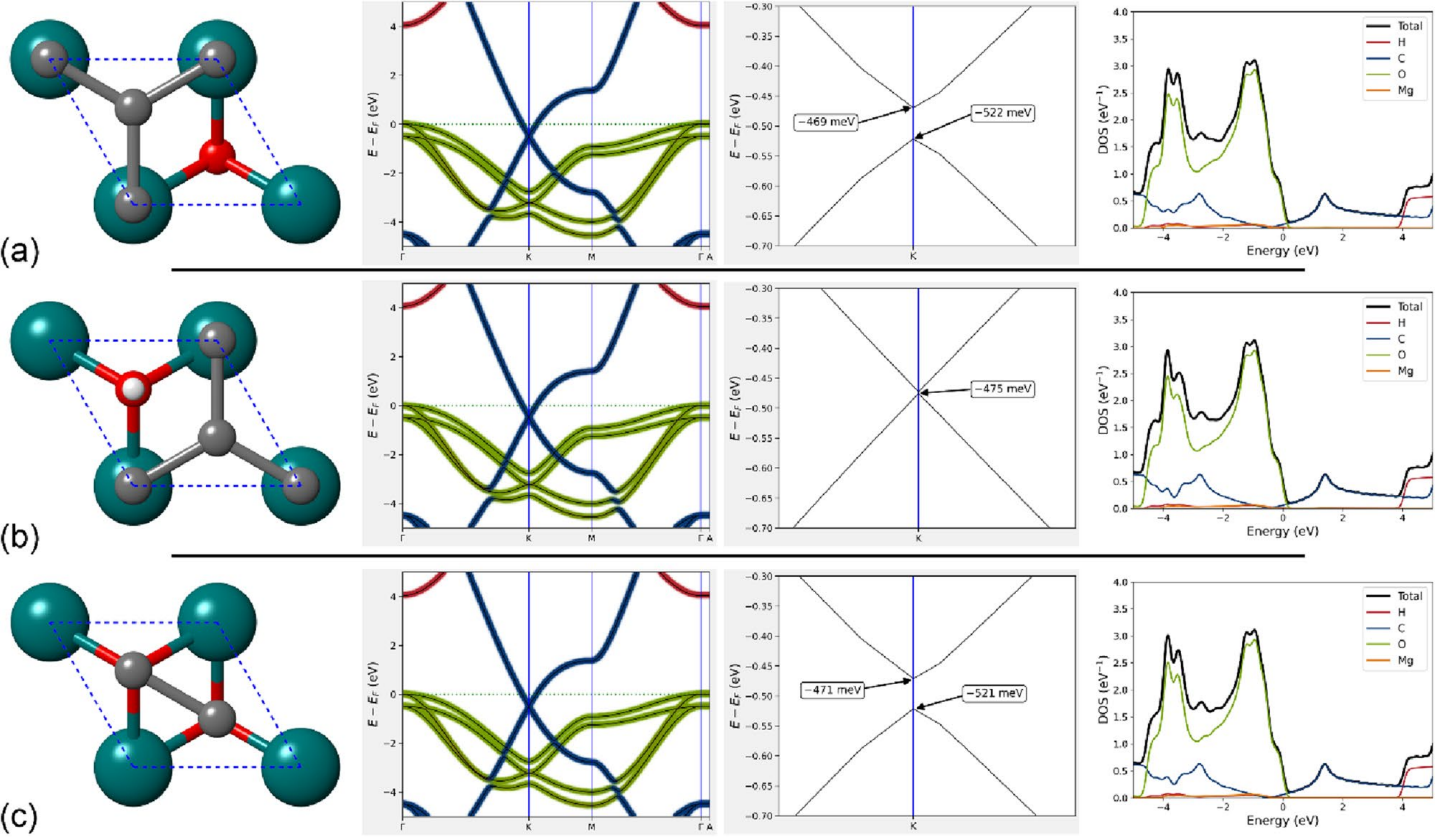
Band structure and density of states of the graphene/brucite interface models. In panels (**a**), (**b**) and (**c**) the results are reported for the BG-ab, BG-ac and BG-bc heterojunctions, respectively. In each panel, from left to right, a ball-and-stick representation of the model, the band structure between ± 5 eV, a zoom on the graphene Dirac point in K and the electronic density of states (total and atomic projections) are reported. The bands are plotted with colours related to the contribution of each element to them (blue—C; orange—Mg; green—O; red—H).

We present in Fig. 3 the ab initio band structure and electron density of states of the three heterojunctions, focused on the region between ± 5 eV. In general, all the electronic bands show the formation of the well-known Dirac cone at the K point in the reciprocal space, which is due to the crossing of the C *p*_*z*_ bands. However, the lowest unoccupied (conduction) band crosses the highest occupied (valence) one about 600 meV below the Fermi level (0 eV). All the other bands of graphene are subject to this energy shift. For example, the C *p*_*x*_ and *p*_*y*_ bands in the composite fall at about – 3.7 eV in the Γ point, whereas in pure graphene they were at about – 3.1 eV. Oxygen *p*_*x*_ and *p*_*y*_ bands transverse the *k-*path between 0 and – 4 eV. All these considerations show the enhanced metallic behaviour of the brucite/graphene heterojunction with respect to pure graphene, which is due to the interaction between the carbon and oxygen orbitals of the two 2D layers, as can be noted also from the projected density of states.

**Figure 4:**
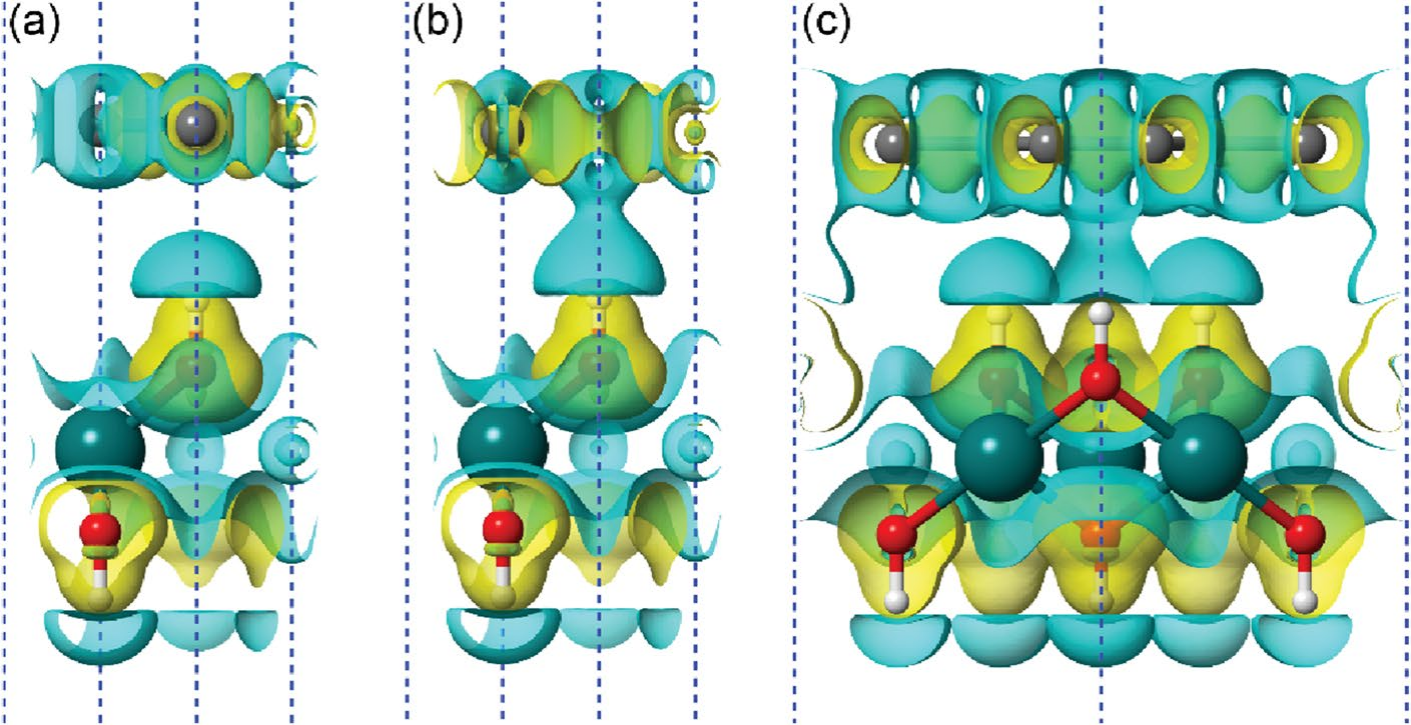
Electron density difference of the brucite/graphene interface models. Isosurfaces of constant electron density difference (at 5.5 × 10^–3^ a.u.) of (**a**) BG-ab, (**b**) BG-ac, and (**c**) BG-L-02 models. Positive and negative density values are represented with yellow and light blue colours, respectively. In panels (**b**) and (**c**) it is possible noting the partial overlap between the electron densities of the brucite substrate and the graphene.

A further interesting feature is the formation of a small gap between the C *p*_*z*_ bands of about 50 meV in BG-ab and BG-bc models calculated with the hybrid HSE06 functional. Conversely, no appreciable gap opening (3 meV) was noted for the BG-ac heterojunction. This is attributable to the perturbative interaction between the electron density clouds of the hydrogen of the hydroxyl group of brucite and the *p*_*z*_ orbitals of graphene, with the maximum band opening when the hydrogen atom is below a C atom (BG-ab and BG-bc models) and the lowest when H is below the hexagonal carbon ring (BG-ac configuration). Figure 4 reports the analysis of the electron density difference of the BG-ab (Fig. 4a) and the BG-ac models (Fig. 4b), the latter showing the partial overlap of the cited electron clouds. We suggest that the observed band opening could be due to a slight localization of the electrons in the BG-ab and BG-bc models, whereas the partial overlap of the electron density clouds of brucite and graphene in the BG-bc system reduces the electron localization.

It is worth noting that these effects are not due to the strain necessarily imposed when constructing the interface between the two layered materials: in fact, from an analysis of the band structure and the Dirac point of pure graphene subject to both compressive and expansive strains (between ± 15%), we noted that (i) the band crossing is always on the Fermi level, (ii) no band gap was formed and (iii), as expected, the band structure expanded in energy by augmenting the stress, and vice versa (see Fig. S2a). Regarding the (001) brucite substrate, no significant variations in the shape of the bands were noticed (Fig. S2b), with the effect of the strain mostly affecting the direct band gap that reduces to 2.145 eV at – 15% (compression) and increases to 3.645 eV at + 15% (expansion). No direct-to-indirect band gap transitions were observed for the magnesium hydroxide.

Since the heterojunction is made from an insulating material (single layer of brucite *E*_g_ = 5.16 eV) and a metal-like one (graphene), it is expected that their interaction could increase or lower the work function *W* of the latter. This energy was calculated for suspended graphene as *W* = 4.529 eV, whereas 4.827 eV, 4.850 eV and 4.828 eV were obtained for the BG-ab, BG-ac and BG-bc models, respectively. All these results were obtained at absolute zero (0 K).

### Medium sized BG-L interface

The purely crystallographic models of the brucite/graphene heterojunction previously discussed showed very interesting electronic features that could help understand the properties of more complex interfaces. In fact, the quite high differences between the lattice parameters of the two materials would not allow a perfect, crystallographic stacking of graphene onto the (001) surface of brucite, as previously discussed. In addition, it is expected that the high strain in the previous models could not be thermodynamically stable without applying stress to the structures because the deformation energy (i.e., the energy required to deform both graphene and brucite to make the perfect coupling) is high, about 200 kJ mol^–1^. Direct transfer of graphene from another substrate (*e.g.*, silicon wafers), or methods such as chemical vapour deposition would probably lead to heterojunctions that are ordered at longer ranges to avoid mechanical strain on brucite, graphene or both. For this reason, we created larger BG models to reduce the imposed strain to both layers, using the previous results to decouple the effects related to different interaction configurations.

**Figure 5:**
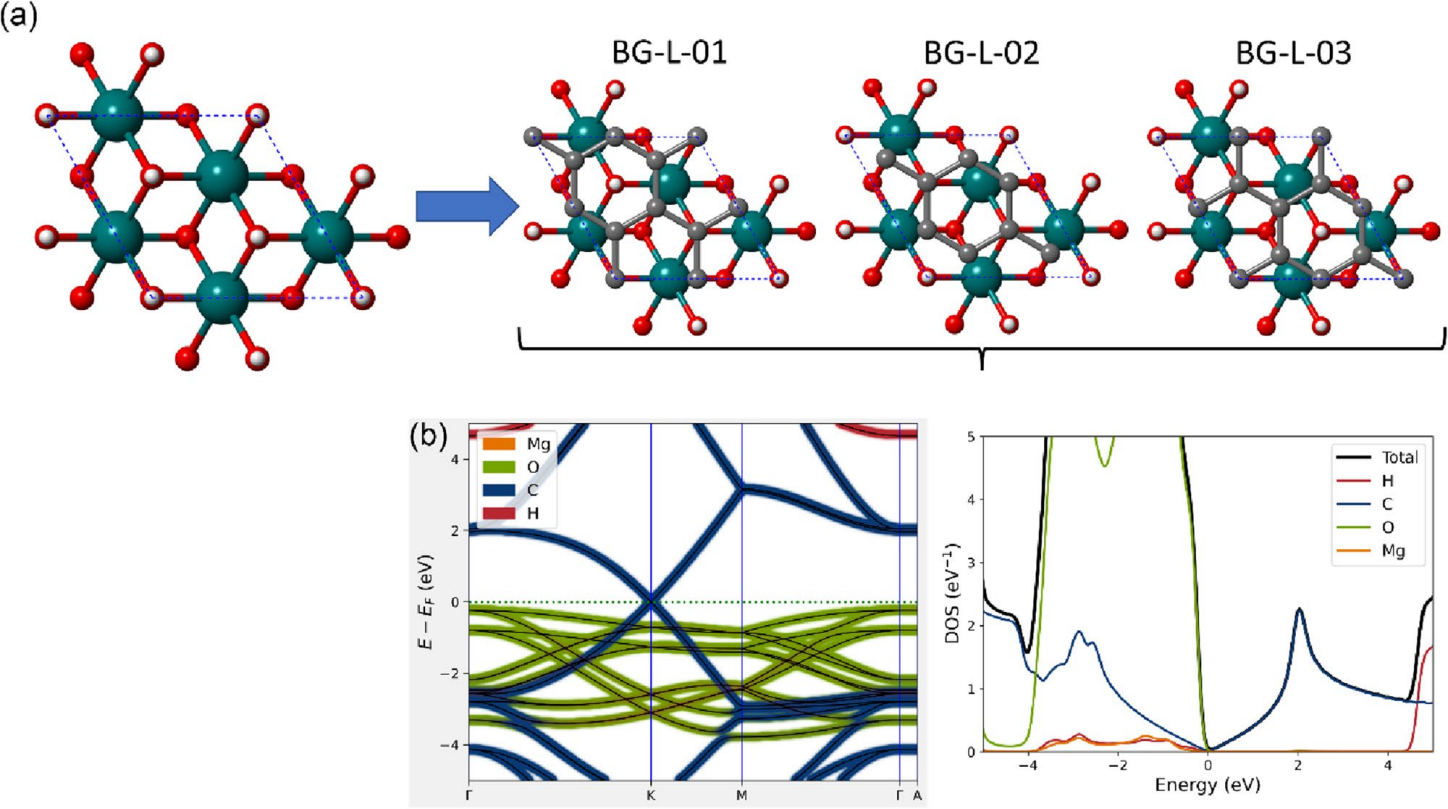
Structural models and electronic properties of the BG-L interface. Panel (**a**) shows the $\sqrt{3}$ × $\sqrt{3}$  × 1 supercell of (001) brucite surface used to realize the BG-L-01, BG-L-02 and BG-L-03 heterojunctions, which are all equivalent in the $P31m$ space group symmetry. Mg, O, H and C atoms are coloured in dark cyan, red, white, and grey, respectively. Panel (**b**) reports the atom-projected, folded band structure of the BG-L heterostructure (on the left) and density of states (on the right) in the ± 5 eV energy range.

To this aim, the interface was modelled from a $\sqrt{3}$ × $\sqrt{3}$ × 1 supercell of (001) brucite surface, with 15 atoms [Mg_3_(OH)_6_, space group $P31m$], and a 2 × 2 × 1 supercell of graphene (8 carbon atoms). The resultant structure, labelled as BG-L, belongs to the $P31m$ space group, with three hydroxyl groups pointing towards the graphene sheet. There are three different ways to place the atomic-flat carbon layer maintaining the most symmetric space group of the material (BG-L-01, BG-L-02 and BG-L-03 models, s.g. $P31m$), which are however equivalent for symmetry reasons (Fig. 5). With these configurations, one hydrogen atom of the brucite like layer is below a hexagonal carbon ring, whereas the other two hydroxyl ions point towards two C atoms. For these models, the mean absolute strain in this brucite/graphene interface is less than 4%, and the binding energy and the distance between the graphene sheet and the (001) brucite surface are ∆*E* = – 17.7 kJ/mol and *d* = 2.440 Å, respectively. This latter distance is very close to the mean distances measured for the BG-ab, BG-ac and BG-bc models, i.e., 2.438 Å. It is worth to be noted that in this case the calculated binding energy takes also into account the deformation energy of the (001) brucite layer and of graphene. In this configuration, the structure results much more stable than the BG-ab, BG-ac and BG-bc models. As expected, after geometry relaxation, all BG-L structures produced the same electronic features, whose band structure and density of states are reported in Fig. 5. In this case, the valence and conduction bands of the *p*_*z*_ C orbitals are at the Fermi level and they do separate in the K point as observed in the smaller BG-ab, BG-ac and BG-bc models. This is due to the interaction between the electron density clouds of the two layers, especially on the site where the hydrogen atom points towards the hexagonal carbon ring, as observed from the electron density difference (see Fig. 4c). As also previously noted, oxygen *p*_*x*_ and *p*_*y*_ bands appear between – 4 and 0 eV. These results indicate that the penetration of the Dirac cone in the valence energy region for the heterojunction made of pure brucite and graphene is due to the combination of the interaction between the single layers and the strain applied to the structure with the penetration increasing with the stress. This could be a very important and controllable variation of the electronic properties that could be very useful, for example, to develop stress and pressure sensors.

### BG-L interfaces with point defects

**Figure 6:**
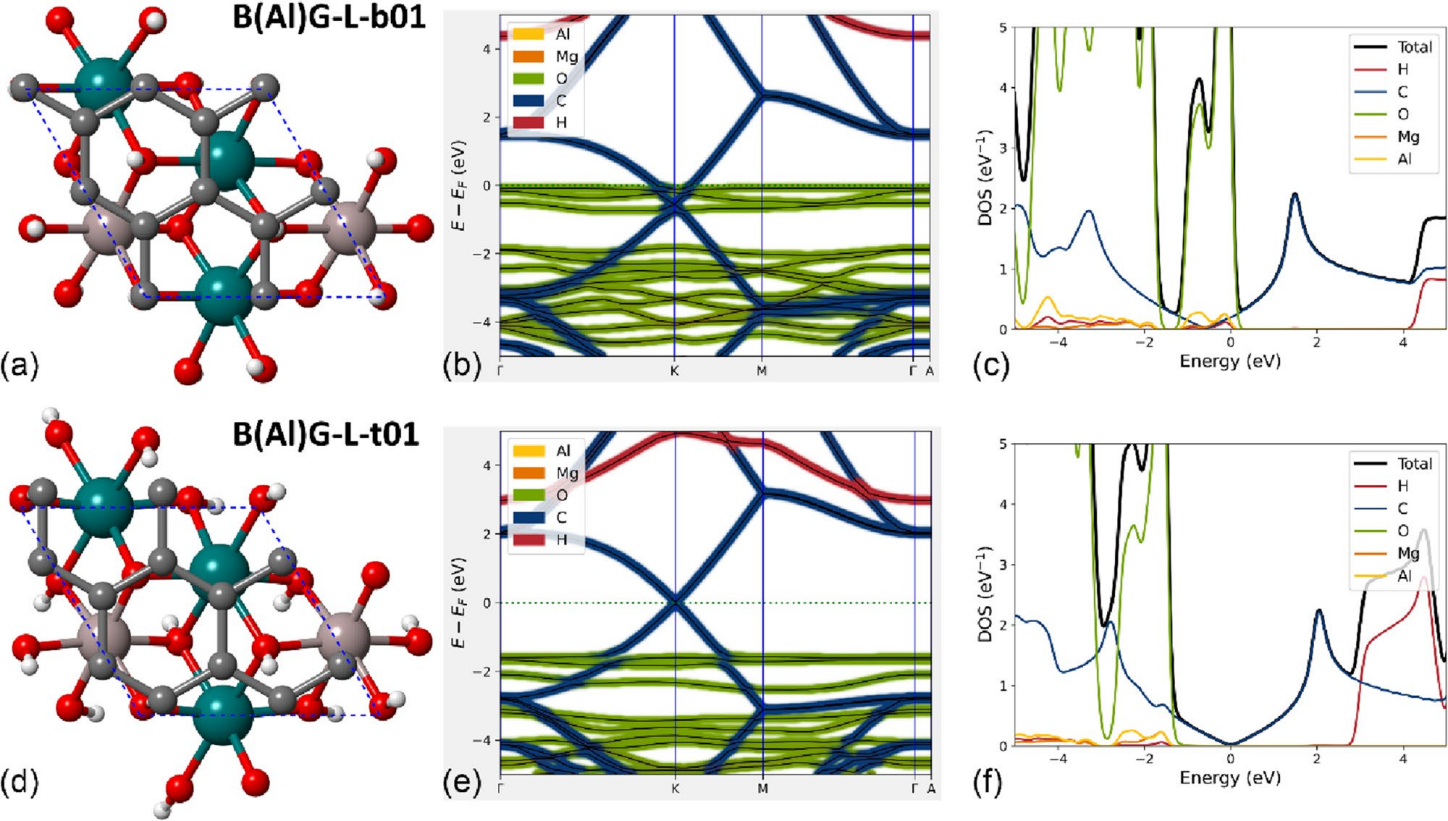
Selected defective brucite/graphene interfaces and related electronic properties. The figure reports the structures of a single graphene sheet interacting with Al-bearing (001) surface of brucite, where a hydrogen atom is absent in the bottom face [B(Al)G-L-b01 model, panel **a**] and in the top face [B(Al)G-L-t01 model, panel **d**]. Mg, Al, O, H and C atoms are coloured in dark cyan, rose, red, white, and grey, respectively, whereas the dashed blue line represents the unit cell. The electronic band structure and the atom-projected density of states of model B(Al)G-L-b01 are reported in panels (**b**, **c**), respectively, whereas the same properties calculated for the B(Al)G-L-t01 model are shown in panels (**e**, **f**).

The $\sqrt{3}$ × $\sqrt{3}$  × 1 supercell of (001) brucite surface is also a suitable substrate to test the effects of point defects on the electronic properties of the BG heterostructure. We chose Al^3+^/Mg^2+^ substitutions because aluminium is a typical substituent of magnesium in natural minerals, such as clinochlore^24^. In addition, the amount of Al^3+^ ions in the so-called brucite-like layer of clinochlore can be, on average, as high as one Al^3+^ every two Mg^2+^ ions, like in the models we realized. Given the $P31m$ symmetry and the 3*c* Wyckoff site (multiplicity 3) occupied by Mg, a single substitution can equivalently occur at any of the magnesium atoms in the model. However, the Al^3+^/Mg^2+^ substitution creates an excess of positive charge in the brucite substrate that must be balanced to ensure the charge neutrality of the system. Among the different ways to restore the zero net charge of the model, in this work, we removed a single hydrogen atom from the (001) brucite surface as the crystal-chemical mechanism to restore the charge neutrality in the layered structure. This surface model was then labelled ‘B(Al)-L’, indicating in parentheses the type of atomic substitution. In previous experimental measurements with Kelvin-probe force microscopy, corroborated by theoretical simulations^28,29^, it was shown that this kind of natural charge balancing in brucite leads to a negative electrostatic potential on the side of the substrate where hydrogen was removed, and consequently to a positive potential on the opposite side (see Fig. S1c,d). Then, in the defective BG heterojunction, the side of the brucite layer exposed to graphene is not equivalent. By our convention, in the present work along the text, we will call ‘top B(Al)-L’ the defective (001) brucite model exposing negative electrostatic potential to graphene, whereas the ‘bottom B(Al)-L’ presents the positive electrostatic potential to the other substrate. Taking into account all possible crystallographic combinations, we modelled six B(Al)G-L structures, three bottom [labelled B(Al)G-L-b01, B(Al)G-L-b02 and B(Al)G-L-b03] and three top [B(Al)G-L-t01, B(Al)G-L-t02 and B(Al)G-L-t03], which have no symmetry elements due to the presence of the point defects (hence, space group $P1$). Some selected results are shown in Fig. 6.

The three bottom models showed negligible variations between each other in terms of both lattice constants and energy difference, with a maximum difference in total energy of about 2 meV. The two layers are held together by a stronger binding energy (– 43.0 kJ/mol) than the stoichiometric BG-L models (– 17.7 kJ/mol), because of the more positive electrostatic surface potential of the brucite substrate, resulting also in a shorter graphene-to-brucite distance (about 2.403 Å). The band gap is absent because, albeit the occupied *p*_*z*_ band and unoccupied *p*_*z*_ band of C are separated by about 20 meV, they penetrate below the Fermi level by about 570 meV. Thus, compared to the BG-L models, the coupled Al^3+^/Mg^2+^ substitution and the H vacancy in the bottom face of brucite increase the penetration of the carbon *p*_*z*_ bands because of the higher positive surface potential of the substrate. In addition, the calculated work function *W* for the three models is between 4.196 and 4.200 eV, which is lower than that of the stoichiometric BG-L structures. Albeit the system is conductive due to the penetration of the *p*_*z*_ bands of carbon below the Fermi energy level, the Dirac cone in the K point is separated by about 20 meV.

A different behaviour was noticed by removing the hydrogen atom of the (001) Al-bearing brucite surface at the interface with graphene (top models). The binding energy of the B(Al)G-L-t01, B(Al)G-L-t02 and B(Al)G-L-t03 systems are slightly lower (about – 35 kJ/mol) than that of the bottom models presented above. Despite the lower interaction energy, the highly negative electrostatic potential due to the Lewis basic site^30^ exposed at the surface of brucite further reduced the distance between this layer and graphene to about 2.300 Å. Very interestingly, the top B(Al)G-L models have very similar electronic band structures, much different from the other simulated systems. In fact, the *p*_*z*_ carbon bands form the Dirac cone at the K point in the first Brillouin zone at the Fermi level, and it was observed a small gap opening of the order of a few tens of meV (between 31 and 45 meV), depending on the model. In addition, after geometry optimization, the B(Al)G-L-t01, B(Al)G-L-t02, and B(Al)G-L-t03, heterojunctions showed a proton disordered structure in the brucite layer induced by the combination of the B/G interactions and the perturbation due to the empty H site. This structure was studied both experimentally^26^ and theoretically^23,31^, and presents a screw-like position of the hydrogen atoms due to a small variation of the Mg–O–H angles. Albeit not perfectly aligned along the **c-**axis, the OH groups in this brucite model are not as canted as in the *α*-quartz case. Furthermore, it is worth to be noted that the surface is still atomic flat and with specific electrostatic potential because of the Al^3+^/Mg^2+^ substitution (see Fig. S1c,d), which are very different from that of the cited silicate.

## Discussion and conclusions

In the present work, we undertook a detailed crystallographic and theoretical exploration of the electronic properties of the interface between graphene and brucite, Mg(OH)_2_, a layered, atomic-flat mineral belonging to the trigonal crystal system. The simulations carried out at the PBE-D2 and HSE06 levels of theory demonstrated that the magnesium hydroxide substrate can deeply alter the band structure of graphene, resulting in a heterojunction with either a more conductive behaviour or a small band gap. The composite is held together by weak van der Waals interactions, with binding energy that increases by including gibbsite-like Al^3+^/Mg^2+^ point defects in the (001) surface of the hydroxide substrate.

Modelling the heterojunction of graphene on brucite would require larger unit cells of both layered materials to reduce the straining to a minimum. Indeed, the strain of about 8% (small models) and 4% (large models) related to the coupling of graphene and Mg(OH)_2_ is high and does not allow at the moment the calculation of other important properties of the heterojunction, for instance electrons and holes mobilities and electron–phonon coupling, quantities that require the lattice dynamics of the system. The phonon band structure calculated for the stoichiometric BG-L heterojunction models (see Fig. S4a) showed a single negative band whose minimum (about – 7 cm^–1^) is located at the centre of the first Brillouin zone (Γ point). This phonon is associated with a sliding motion of graphene over the (001) brucite monolayer, as graphically reported in Fig. S4b. The acoustic modes have the expected null value (0 cm^–1^) in the Γ point, and all the other phonons are positive along the Γ–K–M–Γ–A route in the reciprocal space. Thus, even with a strain of 4% there is only a slight phonon instability in the modelled systems, which would disappear in larger brucite-graphene heterojunction models.

However, the aim of the present investigation was to decouple the effects of the crystallographic orientation of the carbon monolayer with respect to the brucite substrate, to understand how the different interactions affect the band structure of the composite material. The several models here characterized clearly show the possibility to tune the electronic properties according to the crystal-chemistry of the graphene-to-substrate interactions. It was interesting noting that, at absolute zero, the band gap opening in the Al-bearing brucite-graphene interfaces (about 30–45 meV) is one order of magnitude greater than the values calculated for the MoS_2_-1H/graphene heterojunction (about 2.5–2.8 meV)^32,33^. Considering that this band gap is greater than *k*_B_*T* = 26 meV at room temperature, this composite behaves like a semiconductor in standard conditions and a metallic-like behaviour (increased electron conductivity) can be obtained by increasing temperature, hence this material could act as a thermal sensor.

We would like to highlight that brucite is both a naturally occurring mineral and an easily synthesizable phase, for instance from magnesium oxide, MgO (periclase)^34^. Furthermore, the Al^3+^/Mg^2+^ atomic substitutions here considered are commonly found in nature in several minerals. For example, clinochlore may exhibit upon cleavage of the (001) surface intact or remainders of brucite-like layers, which could be suitable as substrates for the creation of heterojunctions. Some studies and applications of this kind of substrate made from clinochlore were reported in relatively recent literature for the adsorption of different (bio)molecules^28–30^. In addition, it was shown the possibility to create nanopatterns at the surface of the cited mineral, e.g., with scanning probes^35^, which could be interesting for the realization of specific graphene/brucite devices with peculiar electronic properties.

All these observations could open to further fundamental research of the physical properties of this kind of natural materials, useful for the design and implementation of innovative heterojunctions with tailored properties and improved features.

The use of PBE-D2 approach proved to be accurate within the approximation introduced in the simulations. In fact, the surface properties of both the stoichiometric and Al-bearing (001) surfaces of brucite are in good agreement with previous simulations at the B3LYP-D* (which include long-range interactions) level of theory and Kelvin probe force microscopy^25,29,30^. In addition, the use of the DFT-D3 correction for long-range interactions, which is more recent and slightly less empirical than the DFT-D2 one here adopted, resulted in structural and electronic features of the heterojunctions that were similar (see Fig. S3 in the Supplementary Materials for an example). The use of the hybrid HSE06 functional, based on the Generalized-Gradient Approximation functional PBE, provided a direct band bap for both the bulk brucite and its (001) single layer, i.e., *E*_g_ = 6.31 eV and *E*_g_ = 5.16 eV, respectively, data in good agreement with the experimental ones obtained for magnesium hydroxide nanodisks (*E*_g_ = 5.70 eV)^36^ and brucite thin films (*E*_g_ = 5.17 eV)^34^. Thus, the electronic properties calculated in this work are accurate and show how the features of the heterojunctions can be easily tuned by the different crystal-chemical properties of the interface (orientation of the single layer, presence of strain and specific point defects), as shown for the opening and penetration of the Dirac cone.

## Methods

### Computational details

All the Density Functional Theory simulations were performed with the QuantumATK code^37^, using the generalized gradient approximation (GGA) functional developed by Perdew, Burke and Ernzerhof (PBE)^38^ and the DFT-D2^39^ and DFT-D3 corrections^40^ to include long-range interactions. This approach was adopted for all structural relaxations. For a more accurate evaluation of the electronic properties of the single layers of graphene and brucite and of their different heterojunctions, the hybrid HSE06 functional^41,42^ was adopted. The crystalline orbitals of the 2D models were described in terms of numerical linear combinations of atomic orbitals (LCAO) basis sets, which employ high-quality double-ζ split valence with polarization functions (DZVP) atomic basis set^43^. The nominal accuracy of the chosen DZVP basis sets is 1.52 meV, according to the results on the ∆-test^44^. After a careful calibration, the first Brillouin zone was sampled on a 47 × 47 × 1 Monkhorst–Pack mesh of 1105 symmetry-reduced *k*-points for the small BG-ab, BG-ac and BG-bc models. For the larger heterojunction models, a comparable accuracy was obtained with a 33 × 33 × 1 *k*-point mesh with 545 irreducible points. The cut-off of the density grid that describes the real-space electron density was set to 200 Ha after carefully checking the convergence on total energy. The electron occupancy of the orbitals was described with the Fermi–Dirac distribution, with a broadening temperature of 300 K. The calculation of the total energy of the models was performed with a self-consistent field (SCF) approach, an iterative procedure that was considered as converged when the energy difference between the last and the previous steps was lower than 10^–7^ eV. Dirichlet (fixed) boundary conditions along the **c** axis were employed for the layered models to properly account for the vanishing potential at the edges of the cell^45^. In this way, the calculation of the chemical potential of the model is equal to the work function of the system under consideration. Calculations for heterojunctions included the counterpoise correction to cancel the basis set superposition error (BSSE)^46^. Phonon band structures were calculated at the PBE-D2 level of theory using the frozen-phonon approach^47^, using a 3 × 3 × 1 supercell containing 207 atoms.

### Modelling details

Initial brucite Mg(OH)_2_ bulk geometry was taken from a previous theoretical work of the authors^23^ and geometrically re-optimized within the selected computational framework. Then, a single layer of Mg(OH)_2_, corresponding to a (001) surface, was cleaved from the bulk and the **c** lattice vector was increased to set a vacuum region of 10 Å above and below the slab. In this way, the replicas of the single layer of brucite along the [001] direction were separated by 20 Å, hence they do not interact with each other. Prior to modelling the brucite/graphene interface, the single layer of Mg(OH)_2_ was geometrically optimized. The same procedure was performed for graphene, which was modelled starting from bulk graphite^48^. For each model, brucite, graphene and their interfaces, the lattice vectors **a** and **b**, and the atomic coordinates were optimized within the same run using the BFGS algorithm, stopping the iterative procedure when the forces and the stress were lower than 10^–3^ eV Å^–1^ and 10^–4^ eV Å^–3^, respectively, to obtain well-converged results.

### Supplementary Information

Supplementary Figures.

## Data Availability

The datasets generated and analysed during the current study are available in the Crystallography Open Database (COD) repository, COD IDs 3000452, 3000453, 3000454, 3000455, 3000456, 3000457, 3000458, 3000459, 3000460, 3000461, 3000462, 3000463.
