# Supplementary material for: Facile band gap tuning in graphene–brucite heterojunctions
Source: Sci Rep. 2023 Dec 28;13:23090. doi: 10.1038/s41598-023-50037-z (PMC10754930; doi:10.1038/s41598-023-50037-z)
Supplement: Supplementary file 1 — Supplementary Figures. [file 41598_2023_50037_MOESM1_ESM.docx]

**Facile band gap tuning in graphene-brucite heterojunctions**

Gianfranco Ulian^1^, Giovanni Valdrè^1,^*

^1^Dipartimento di Scienze Biologiche, Geologiche e Ambientali, Centro di Ricerche Interdisciplinari di Biomineralogia, Cristallografia e Biomateriali, Università di Bologna “Alma Mater Studiorum” Piazza di Porta San Donato 1, 40126 Bologna, Italy.

**Supplementary Figures/Tables**


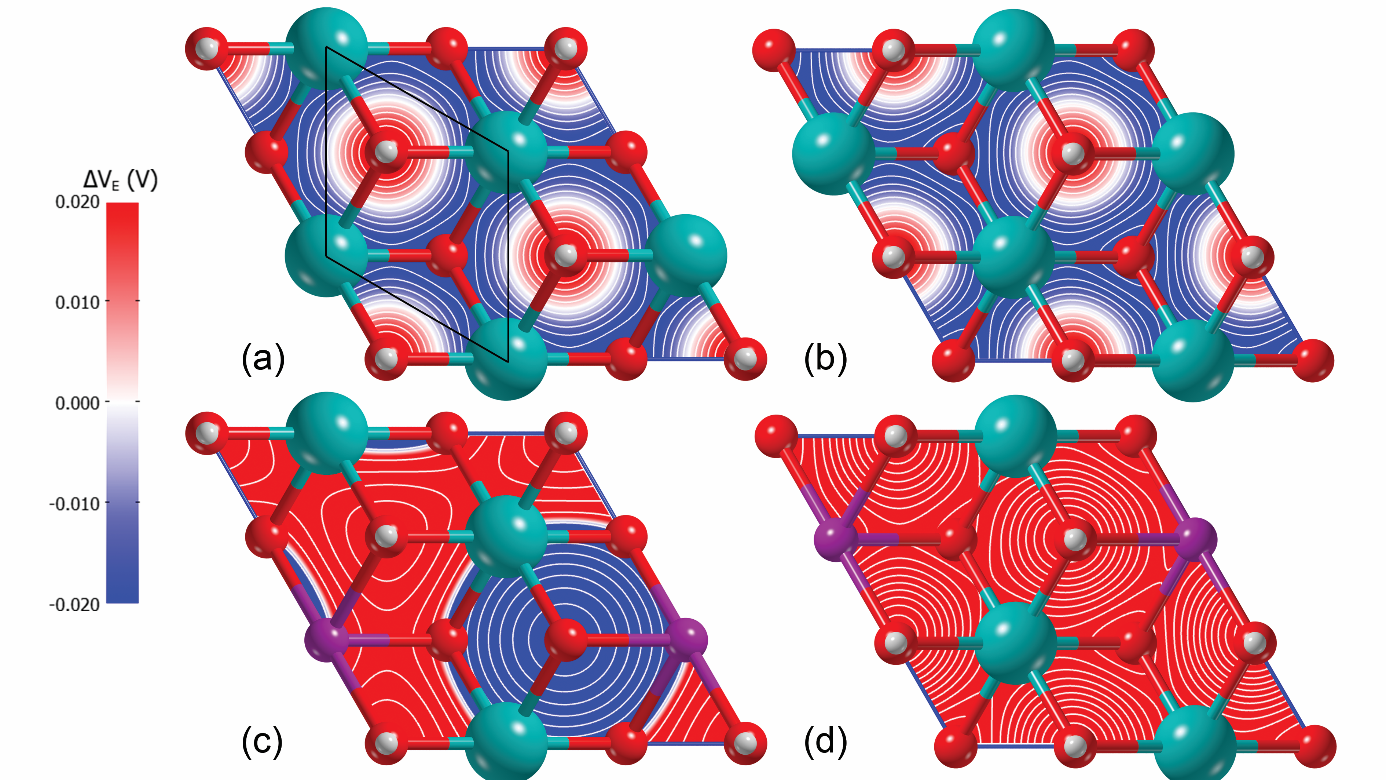


**Fig. S1 Electrostatic potential of stoichiometric and Al-bearing (001) brucite surfaces.** Panels (a,b) show the electrostatic potential maps of the top and bottom surfaces of the $\sqrt{3}$ × $\sqrt{3}$ × 1 supercell model of pure (001) brucite, respectively. Conversely, panels (c,d) report the electrostatic potential features of the top and bottom surfaces of the B(Al)-L models, respectively. The maps were calculated at 2 Å from the topmost atom of the surface model. The unit cell model was superimposed to the map to better correlate the surface potential to the crystal-chemistry. Mg, Al, O and H atoms were coloured in dark cyan, purple, red and white, respectively. The black lines in panel (a) show the 1 × 1 × 1 unit cell of (001) brucite surface.


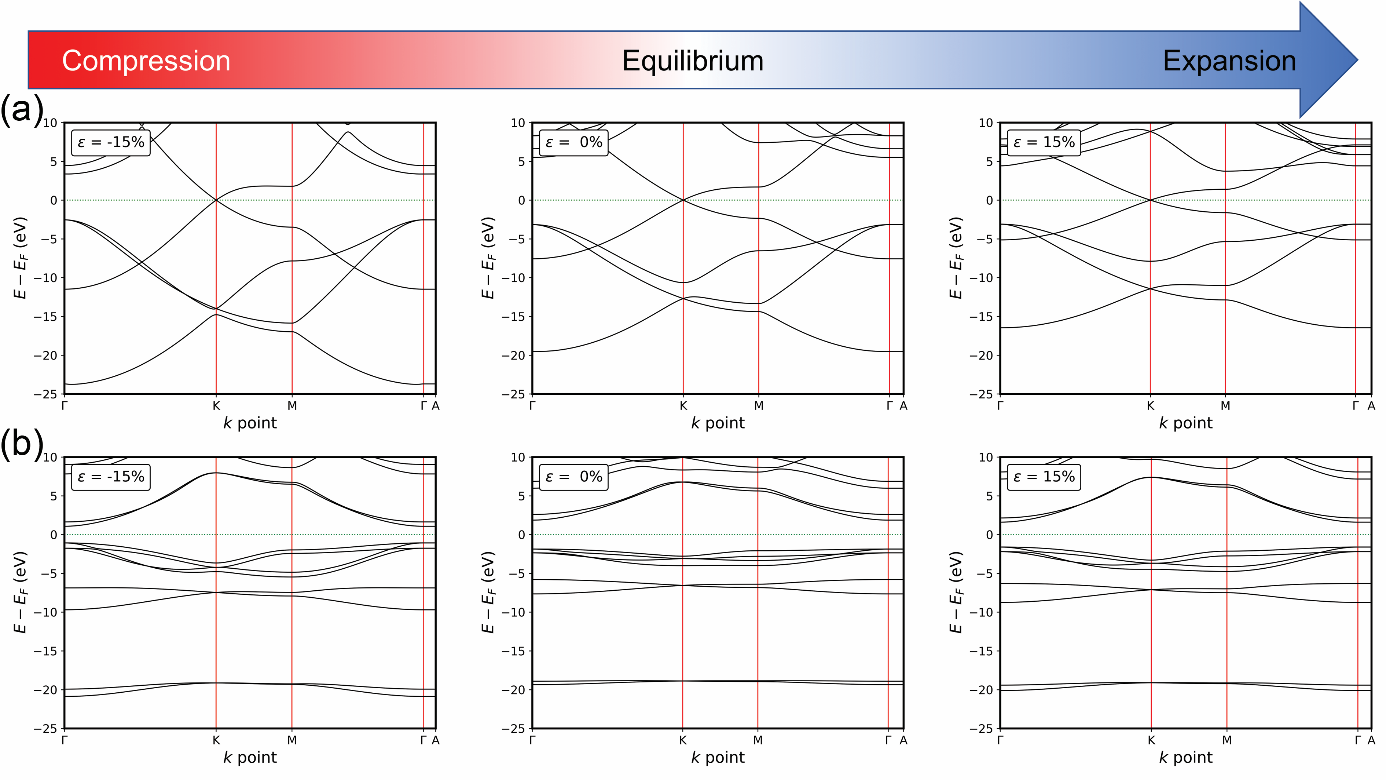


**Fig. S2 Effect of strain *ε* on the electronic band structure of layered materials.** The band structure of (a) graphene and (b) (001) brucite surface was calculated along the *k*-path Γ-K-M-Γ-A between ±15% the *a* lattice parameter value of the single layers (negative values mean compression, positive ones expansion of the unit cell). Note in panel (a) that the Dirac point (crossing of the valence and conduction bands) at K is not affected by elastic deformation of graphene.


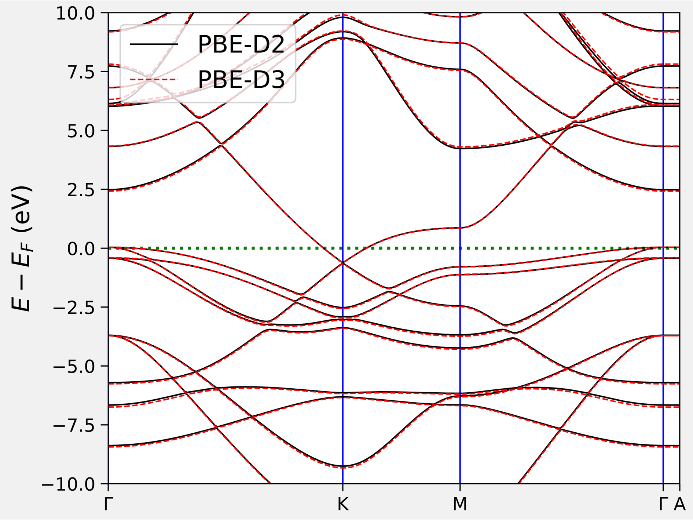


**Fig. S3 Effect of different methods to include long-range interactions on the electronic band structure of the heterojunctions.** The band structures here reported are related to the BG-ab heterojunction (see text for details), as obtained from the structure optimized with the DFT-D2 (black continuous lines) and DFT-D3 (red dashed lines) corrections.


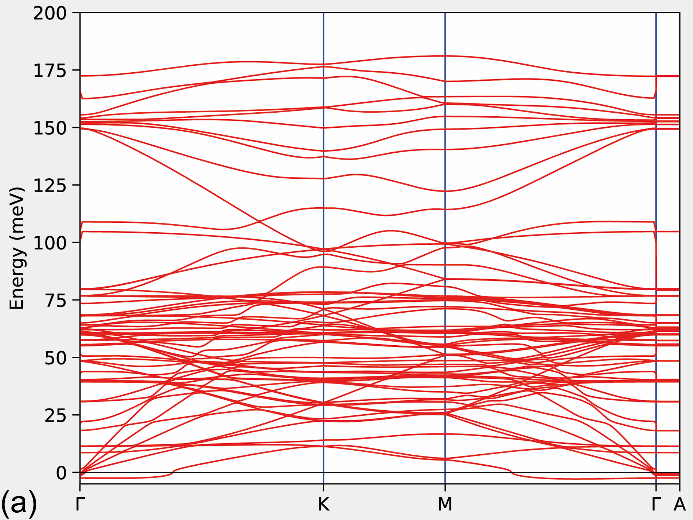

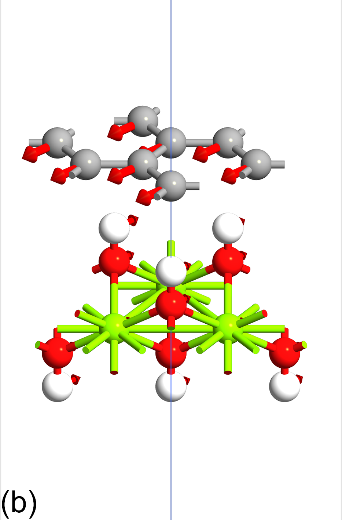


**Fig. S4 Phonon band structure of the BG-L heterojunction models.** All the bands shown in panel (a) are positive at the centre of the first Brillouin zone (Γ) but one. This negative mode falls at about –7 cm^–1^, and its associated atomic vibration is shown in panel (b), where it is possible to note the sliding mechanism of one layer over the other (see the red arrows). The phonons were calculated with a 3×3×3 supercell at the PBE-D2 level of theory.
